# Transcriptome Profiles Reveal the Crucial Roles of Hormone and Sugar in the Bud Dormancy of *Prunus mume*

**DOI:** 10.1038/s41598-018-23108-9

**Published:** 2018-03-23

**Authors:** Zhiyong Zhang, XiaoKang Zhuo, Kai Zhao, Tangchun Zheng, Yu Han, Cunquan Yuan, Qixiang Zhang

**Affiliations:** Beijing Key Laboratory of Ornamental Plants Germplasm Innovation & Molecular Breeding, National Engineering Research Center for Floriculture, Beijing Laboratory of Urban and Rural Ecological Environment, Key Laboratory of Genetics and Breeding in Forest Trees and Ornamental Plants of Ministry of Education, School of Landscape Architecture, Beijing Forestry University, Beijing, 100083 China

## Abstract

Bud dormancy transition is a vital developmental process for perennial plant survival. The process is precisely regulated by diverse endogenous genetic factors and environmental cues, but the mechanisms are not yet fully understood. *Prunus mume* is an ideal crop for bud dormancy analysis because of its early spring-flowering characteristics and small sequenced genome. Here, we analyzed the transcriptome profiles at the three endodormancy stages and natural flush stage using RNA sequencing combined with phytohormone and sugar content measurements. Significant alterations in hormone contents and carbohydrate metabolism have been observed, and α-amylases, Glucan Hydrolase Family 17 and diphosphate-glycosyltransferase family might play crucial roles in the interactions between hormones and sugars. The following hypothetical model for understanding the molecular mechanism of bud dormancy in *Prunus mume* is proposed: low temperatures exposure induces the significant up-regulation of eight *C-repeat binding factor* genes, which directly promotes all six *dormancy-associated MADS-box* genes, resulting in dormancy establishment. The prolonged cold and/or subsequently increasing temperature then decreases the expression levels of these two gene families, which alleviates the inhibition of *FLOWERING LOCUS T* and reopens the growth-promoting pathway, resulting in dormancy release and the initiation of the bud break process.

## Introduction

Perennial plant species face a multitude of abiotic stresses during their long lifespans. In winter, low temperature and short photoperiod are the most serious threats to most tree species in temperate zones^1^. Bud dormancy is a bet-hedging strategy for avoiding injury in unsuitable environments and for synchronizing their annual growth. Furthermore, bud dormancy is an agronomically important trait, influencing flower uniformity and fruit quality in the following growing season^2^. Lang *et al*.^3^ distinguished three types of bud dormancy: paradormancy, mainly promoted by other plant organs; endodormancy, maintained by signals internal to the bud; ecodormancy, induced by external environmental factors. Dormancy is regulated by complex endogenous signaling networks, such as hormone signaling and sink/source organ activity, and multiple environmental signals, such as temperature and day length, to ensure flowering at right time^4^. In recent years, dormancy regulation has garnered additional interest owing to global warming and unseasonal temperature fluctuations^5^.

Recently, increasing transcriptomic studies have been carried out to define gene regulatory networks in buds during the dormancy transition process, and several conserved factors been identified. An important breakthrough was the determination of the close relationship between *dormancy-associated MADS-box* (*DAM*) genes and dormancy phase transition, up-regulated during dormancy induction and down-regulated during release^6^. This seasonal expression patterns are reported in peach^7–10^, pear^11^, apple^12,13^, Japanese pear^14^ and Japanese apricot^15^. Recently, Niu *et al*.^16^ constructed a proposed *DAM* centered model in pear. The low temperatures in autumn promote the accumulation of *C-repeat binding factor* (*CBF*), which can directly promote *DAM* expression. Subsequently, the increased *DAM* inhibits *FLOWERING LOCUS T* (*FT*) expression, which can induce the endodormancy establishment. After enough chilling achieved, miR6390 promotes the degradation of *DAM*s mRNAs to promote endodormancy release. Although an increasing number of studies have focused on this field and achieved some significant achievements, the genetic factors underlying the control of dormancy are still not well understood.

The crucial roles of phytohormones in bud dormancy regulation are becoming more evident^17^. It is well known that abscisic acid (ABA) and gibberellins (GAs) play antagonistic roles in bud dormancy^18–20^. A high level of ABA increases the depth of dormancy, while a high level of GA increases the release of dormancy. ABA levels may increase in the autumn, resulting in the inhibition of cell proliferation and the induction of dormancy. The participation of GAs in breaking poplar bud dormancy has been well reported^21^. GA induces the up-regulation of 1,3-β-glucanases to hydrolyze callose at the plasmodesmata (PD), providing conduit for *FT* transport to promote dormancy release process^22,23^. In fact, not only endogenous ABA/GA individual concentrations, but also a dynamic balance of ABA/GA hormone-signaling network is thought to be central to dormancy induction and release^20,24–26^. In addition, recent findings demonstrate that other hormones, such as indole-3-acetic acid (IAA), ethylene (ET), cytokinin (CK), salicylic acid (SA), jasmonic acid (JA), and brassinosteroids (BR), are also critical for inducing and maintaining dormancy and, therefore, might act as key protectors of seed dormancy^27^. The interactions among these plant hormones controls the interconnected molecular process of bud dormancy, but their detailed roles remain to be discovered.

Tarancón *et al*.^4^ proposed that dormant buds are in carbon starvation situation triggered by environmental and endogenous cues. In the face of this carbon-limiting situation, the plants adjust carbohydrate metabolism between storage and soluble sugars to support essential maintenance functions^28,29^. For example, total soluble sugars increase at the onset of cold conditions, reaching their maximum at full cold hardiness and declining during de-acclimation^30^. Additionally, sucrose may be accumulated to protect against cold-induced damage under winter’s low-temperature conditions^31^. Recently, energy metabolism was demonstrated as being essential for dormancy transition, not only as energy substance but also as sugar-related signals^32–34^. While the role of sugar as an energy supply has been relatively well studied, the possible roles in mediating bud dormancy transition are still largely unknown.

*Prunus mume*, a rosaceous tree, is highly valued for its ornamental and economic importance. The most striking distinction of *P. mume* flowers is the early spring-flowering^35^. These flower buds continue to develop until late autumn, and then enter into dormant stage triggered by short daylength and low temperature. After passing a certain period of chilling temperature, flowers resume growth once environmental conditions are suitable^36^. During the long breeding time, the availability of diverse germplasm having different chilling requirements and flowering times provided excellent biomaterials for bud dormancy analyses. Previous transcript studies of dormancy release for several *Prunus* genus, such as *P. avium*^37,38^, *P. armeniaca*^39^ and *P. persica*^40–43^ have been performed, and some important regulators and signaling pathways have been identified. However, the gene regulatory networks are not well defined. Furthermore, the conservation of these identified genes throughout tree species is not well established^4^. Thus, understanding how *P. mume* perceives these seasonal changes to orchestrate the genetic regulation is critical, especially in the context of climate unseasonal change.

Endodormancy differs from the other types of dormancy. Endodormant buds cannot resume growth under favorable conditions, and require enough chilling accumulation for the transition to ecodormancy. Genes showing chilling-mediated differential expression patterns are candidates for internal factors controlling endodormancy. In this study, we further separate endodormancy into three stages (EDI, EDII and EDIII) according to chilling accumulation, and a natural flush (NF) stages were also taken into consideration during the analysis of the entire dormancy-activity cycle using RNA sequencing (RNA-Seq). Additionally, the sugars and hormones contents were measured using a spectrophotometer and high-performance liquid chromatography coupled to electrospray ionization–tandem mass spectrometry (HPLC–ESI–MS/MS). These novel transcriptomes offer comprehensive expression profiling data for a dynamic view of gene variations during bud dormancy in *P. mume*.

## Results

### Quantification and measurement of bud dormancy status in *P. mume*

**Figure 1:**
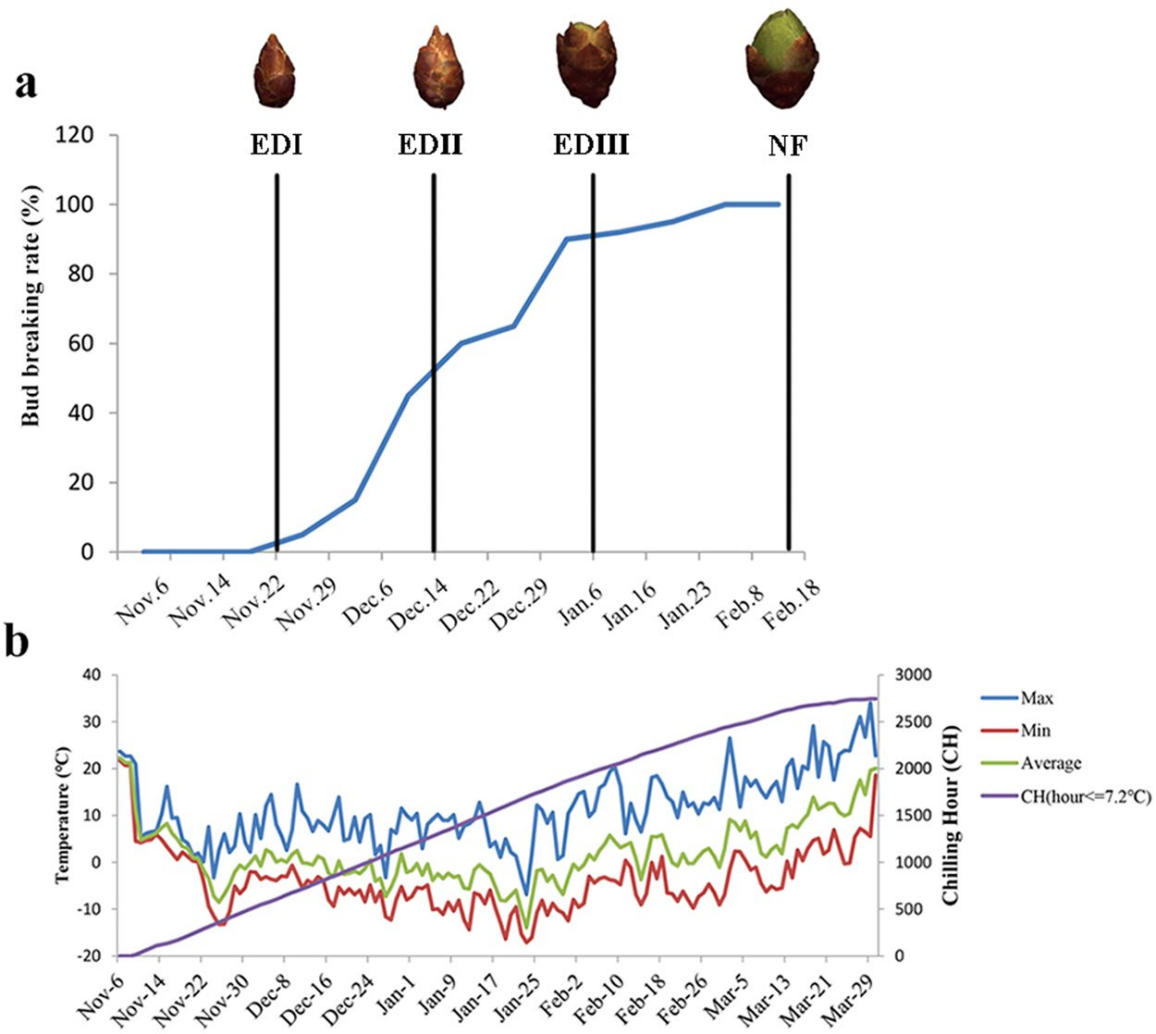
Characterization of flower bud dormancy status and seasonal temperature records. (**a**) Changes in bud dormancy status represented by bud-break percentages after 10 d phytotron cultivation and morphologic features at four representative time points. (**b**) Daily maximum, minimum and average temperatures and cumulative chilling hours during the sample collection periods.

Until now, quantifying the exact dormancy status has been a prerequisite of dormancy studies, and no convincing method nor validated molecular marker have been available for demarcating the status^18^. In this study, we measured the dormancy level using repeated samplings of branch cuttings as detailed in the Methods section. Figure 1a shows the percentage of bud breaking after 10 d of controlled cultivation in a phytotron, and morphologic features at four representative sampling points. The chilling accumulation (number of hours below 7.2 °C) was recorded using a HOBO (Fig. 1b). On Nov. 6 [accumulated chilling hours (CH) = 0], leaves began to fall, and flower buds had no signs of flushing under controlled conditions. By Nov. 22, the accumulated chilling hours achieved 248 CHs, the one-year-old shoots still had no flush sign in the phytotron. Thus, we termed this time point as endodormancy I (EDI). From Nov. 29, the flower buds started to be released (~3%). However, the flush rate was negligible and unstable among the three replicates, and the so-called flushed buds were aborted immediately. On Dec. 6, with a 15% flush rate and 605 CHs, buds fluctuated depending on the cuttings. Moreover, the flushed buds were mainly at the middle position of the shoots, indicating different dormancy states even in the same shoot. By Dec. 14, there was a 45% flush rate and 748 CHs. The flush rate was stable. Therefore, we termed this time point as endodormancy II (EDII). From Dec. 14 to Jan. 6, bud-burst percentage continued to increase, and time to bud burst continued to decrease. By Jan. 6, the flower buds had completely flushed, and, more importantly, all of the flushed flowers were morphologically normal after a 6-d controlled cultivation in a phytotron. Therefore, we termed this time point as endodormancy III (EDIII). On Feb. 18, flower buds showed green tips under natural conditions, suggesting that dormancy had been completely released. Thus, based on morphology and chilling accumulation, we considered Nov. 22 as EDI, Dec. 14 as EDII, Jan. 6 as EDIII and Feb.18 as NF.

### Hormone contents in buds during dormancy transition

**Figure 2:**
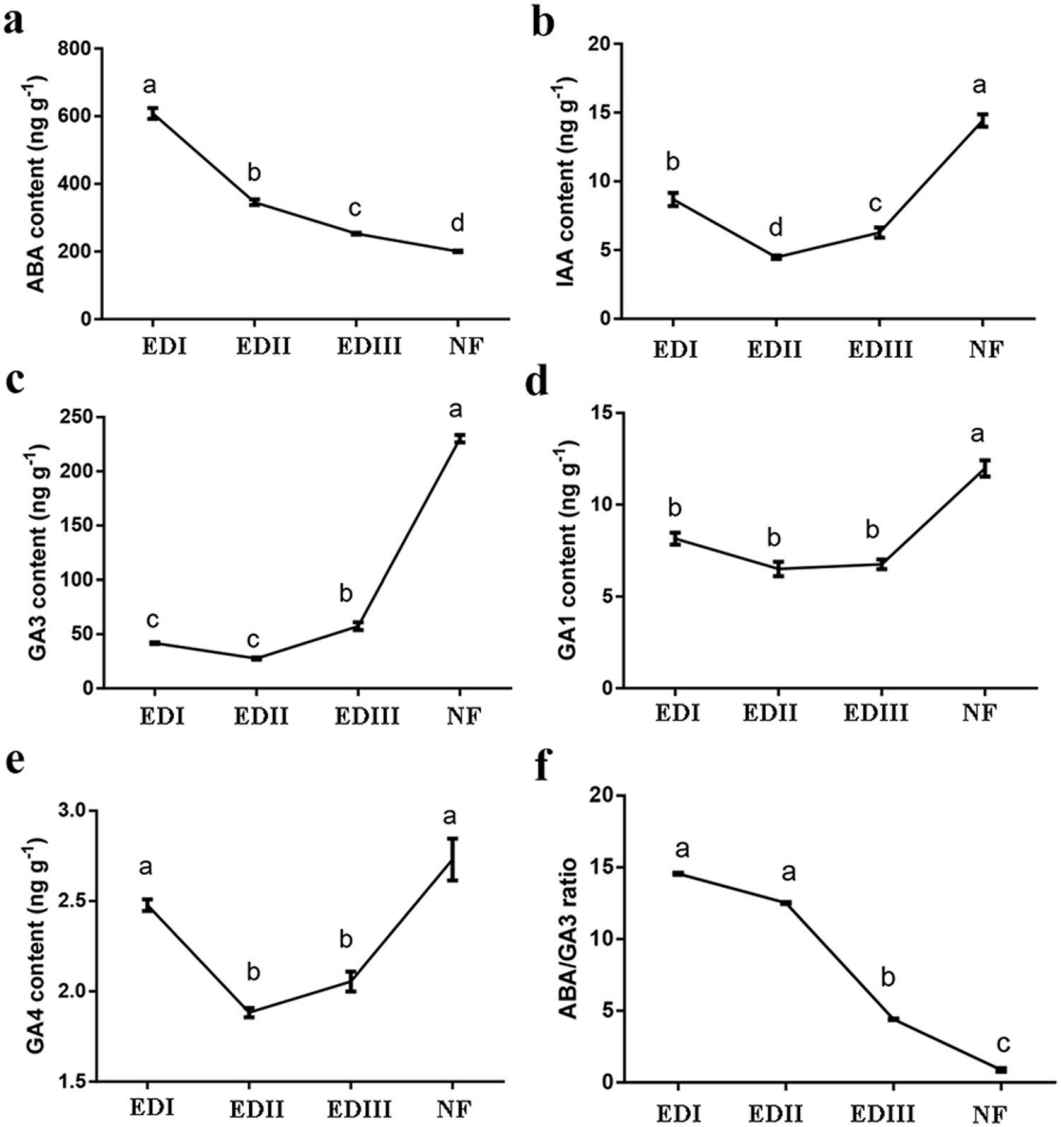
Phytohormone content in flower buds during the dormancy process in *P. mume*. (**a**) ABA; (**b**) IAA; (**c**) GA3; (**d**) GA1; (**e**) GA4; (**f**) ABA/GA3 ratio. Values are means of three replicates ± SE. Data are means with three biological replicates each, with error bars representing standard error. Non-overlapping letters (**a**–**d**) indicate significant differences between different bud stages, based on analysis of variance and multiple range test procedures with a confidence level of 95%.

The levels of hormones were analyzed at four dormant points by HPLC–ESI–MS/MS (Fig. 2). The ABA content was high in EDI, decreased sharply by 44% from EDI to EDII and then by 27% from EDII to EDIII and 21% from EDIII to NF (Fig. 2a). The IAA content decreased by 48.5% from EDI to EDII but then increased sharply by 40% from EDII to EDIII and 130% from EDIII to NF (Fig. 2b). To our surprise, a consistent trend was observed for the IAA and GA contents. The GA3 content was relatively low during the EDI, EDII and EDIII dormancy stages but then increased sharply by 303.5% from the EDIII to NF stages (Fig. 2c). This trend was also observed for the GA1 and GA4 contents, although their levels were very low (Fig. 2d,e). Interestingly, the ABA/GA3 ratio was observed with little changes between EDI and EDII, and then decreased sharply by 64.1% from EDII to EDIII and 81.8% from EDIII to NF (Fig. 2f).

### Sugar contents in buds during dormancy transition

**Figure 3:**
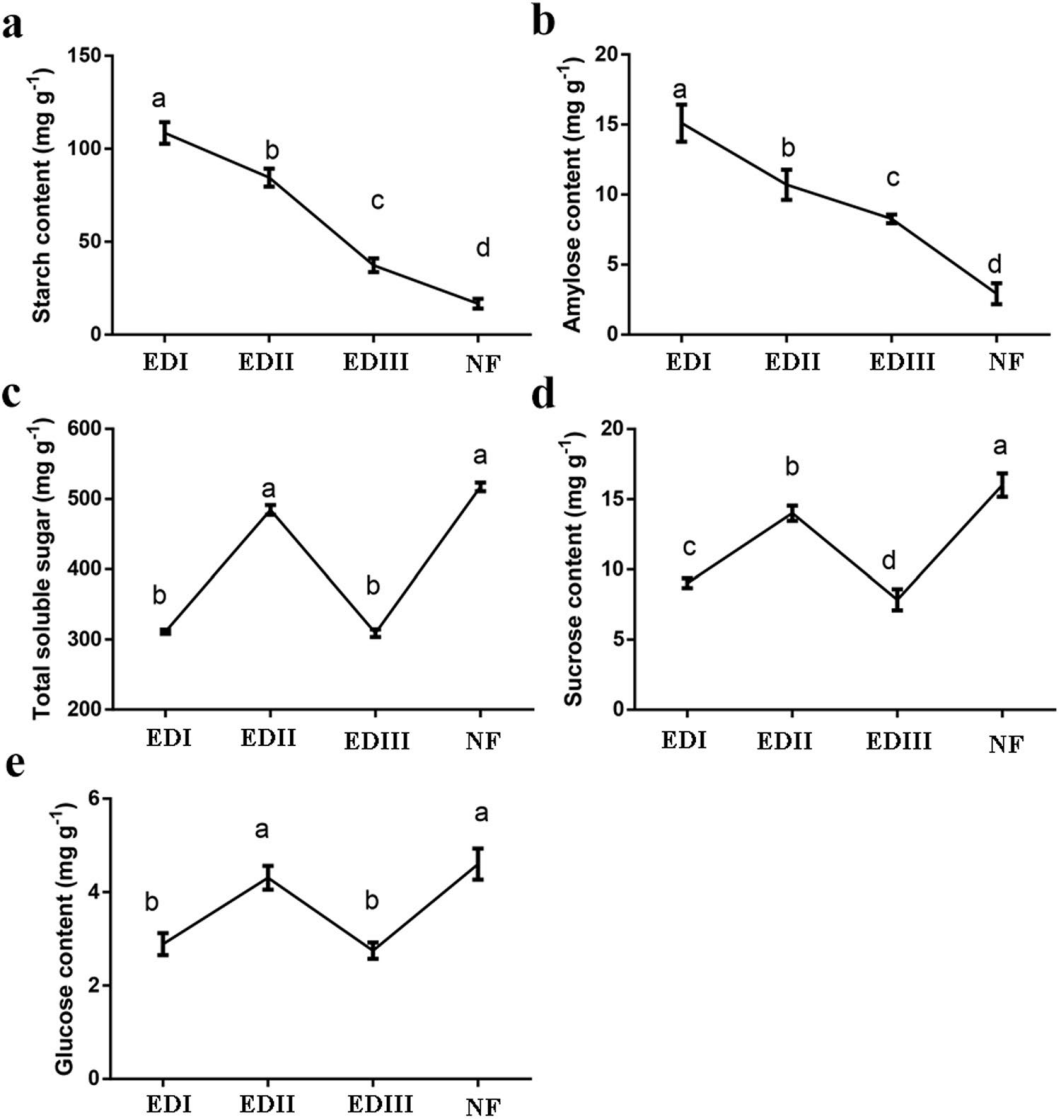
Sugar content in flower buds during the dormancy process in *P. mume*. **(a)** starch; **(b**) amylose; (**c**) total soluble sugar; (**d**) sucrose; (**e**) glucose. Values are means of three replicates ± SE. Data are means with three biological replicates each, with error bars representing standard error. Non-overlapping letters (**a**–**d**) indicate significant differences between different bud stages, based on analysis of variance and multiple range test procedures with a confidence level of 95%.

The contents of sugars and their catabolites were also analyzed at the four time points (Fig. 3). Starch and amylose levels were high at the beginning of bud dormancy, and then decreased significantly from EDI to NF stages (Fig. 3a,b). Soluble sugar, sucrose and glucose increased significantly from the EDI to EDII stage and then decreased sharply from the EDII to EDIII stage, peaking during the EDII stage (Fig. 3c,d and e). The contents of soluble sugars then increased by 138.0% from the EDIII to NF stage, suggesting that a considerable amount of energy was needed to support flower flush in early spring. The sucrose and glucose contents underwent similar change trends, increasing dramatically by 104.5% and 65.5%, respectively, from the EDIII to NF stage.

### Global analysis of RNA-Seq Data

To obtain a genome-wide view of the transcriptome changes in the dormancy transition progression of *P. mume*, four developmental stages were constructed using RNA-Seq. After stringent quality checks and data trimming, a total of 438,914,500 high quality clean reads containing 65,837,175,000 nucleotides was obtained (Table 1). The percentages of Phred quality scores ≥20 at each base position were all greater than 98%. More than 84% of high quality reads from individual samples could be mapped on the reference genome, and 24,212 (out of 31,390 predicted genes) genes were found to be expressed (data not shown). More details are shown in Table 1. All of the raw read data were deposited in the Genome Sequence Archive with the project ID PRJCA000291.

**Table 1:** A summary of the transcriptome changes at four dormancy stages with three biological replicates.

Samples	Reads Number	Bases(bp)	GC(%)	Q20(%)	Total Mapped Reads	Mapped Pair Reads
EDI-1	36144152	5421622800	46.11	98.12	84.00%	75.40%
EDI-2	36867980	5530197000	45.56	98.25	86.10%	78.30%
EDI-3	35877484	5381622600	45.67	98.29	86.10%	78.40%
EDII-1	36525008	5478751200	45.28	98.11	86.20%	78.20%
EDII-2	36810818	5521622700	45.50	98.03	85.70%	77.30%
EDII-3	36400070	5460010500	45.79	98.25	84.60%	76.80%
EDIII-1	37191674	5578751100	45.14	98.06	86.10%	77.90%
EDIII-2	36543384	5481507600	44.89	97.98	85.20%	76.40%
EDIII-3	36497230	5474584500	45.36	98.05	84.20%	75.50%
NF-1	36690996	5503649400	45.81	98.34	86.50%	79.20%
NF-2	36174030	5426104500	45.69	98.25	85.90%	78.50%
NF-3	37191674	5578751100	45.77	98.25	85.90%	78.40%

### Overall relatedness among the 12 transcriptomes

The degree of reproducibility was evaluated based on the square of the Pearson correlation coefficient, represented by the R^2^ value. A principal component analysis (PCA) was also performed to estimate the overall relatedness of transcriptomes. Overall, the expressions between all of the biological replicates were highly correlated, and this dataset could be used for further statistical analyses. Additionally, the R^2^ values of EDI *vs* EDII and EDII *vs* EDIII were high, and the R^2^ values between replicates of NFs were relatively low (data not shown). The PCA result was consistent with the analysis of the Pearson correlation coefficient (Supplementary Fig. S1). These data suggested that the dormancy release was a smooth transition process, and that the NF was a stage of dramatic changes with a more complex transcriptome.

### Successive pairwise comparisons of DEG profiles

Because of the smooth dormancy transition, we compared transcriptomes of each successive stage with false discovery rates ≤0.05 and fold changes ≥1.5 and fold change ≤−1.5 (|log_2_Ratio| ≥0.58496). In total, 5,831 DEGs were found to be significantly changed during the dormancy transition process (Supplementary Table S1). Among these, 1,198 DEGs occurred between the EDI and EDII libraries, of which 515 were up-regulated and 683 were down-regulated. Between EDII and EDIII, there were 1,116 DEGs, with 403 up-regulated and 713 down-regulated. As expected, the largest number, 4,751 DEGs, was found between EDIII and NF (1,749 up- and 3,002 down-regulated), indicating that the transcript changed dramatically at these key stages. The numbers of DEGs in EDIII *vs* NF was greater than in EDI *vs* EDII or EDII *vs* EDIII, indicating the involvement of complex developmental events during NF. We also analyzed the overlap between the pairwise comparisons at both stages as shown in a Venn diagram (Supplementary Fig. S2). A total of 4,787 (82.1%) transcripts exhibited developmental stage-specific differential expressions, and only a small fraction of genes (190, 3.3%) were found to be common in all the comparisons. A large proportion of DEGs (392), overlapped between EDI *vs* EDII and EDII *vs* EDIII, suggesting a relatively smooth transition at the EDI-EDII-EDIII.

### GO, KEGG and MapMan annotation analyses of all DEGs

DEGs were annotated by GO, KEGG and MapMan analyses to examine putative functional differences between different successive developmental stages. The significantly enriched GO terms (P-value < 0.05 FDR corrected) were involved in the cellular location, molecular function and biological processes (Supplementary Fig. S3). EDI vs EDII and EDII vs EDIII had far fewer GO terms than EDIII vs NF. Surprisingly, the GO term “microtubule-based process” was only identified in the EDI vs EDII cluster, and “hormone transport” was only identified in the EDII vs EDIII cluster. The terms “response to hormone” and “metabolism” were significantly enriched only in the EDIII vs NF comparison (Supplementary Table S2). The DEGs were then subjected to a KEGG pathway mapping, and the top 20 enriched pathways in each comparison are shown in Supplementary Fig. S4. KEGG showed the dramatic changes in the metabolism of various hormones and starch and sucrose. In addition, several other metabolic pathways, such as biosynthesis of secondary metabolites and secondary cell wall, were also significantly represented (Supplementary Table S3). The MapMan BINs analysis was also used to reveal a global view of changes in several important metabolic pathways and related functional groups during the dormancy transition process. Metabolic activities within the bud are significantly down-regulated during dormancy and then reactivated during release (Supplementary Fig. S5a–c). These effects and changes were mainly in “glycolysis”, “sucrose degradation”, “cell wall” and “lipid metabolism”. In corroboration with previous studies, we found that the DEGs participating in several hormone metabolism and signaling pathways, like ABA, GA, IAA, ET, JA, SA and BR were significantly changed (Supplementary Fig. S5d–f; Supplementary Table S4). Overall, the results were consistent with our enriched GO and KEGG pathways.

### Cluster analysis of hormone-regulated DEGs during bud dormancy transition

**Figure 4:**
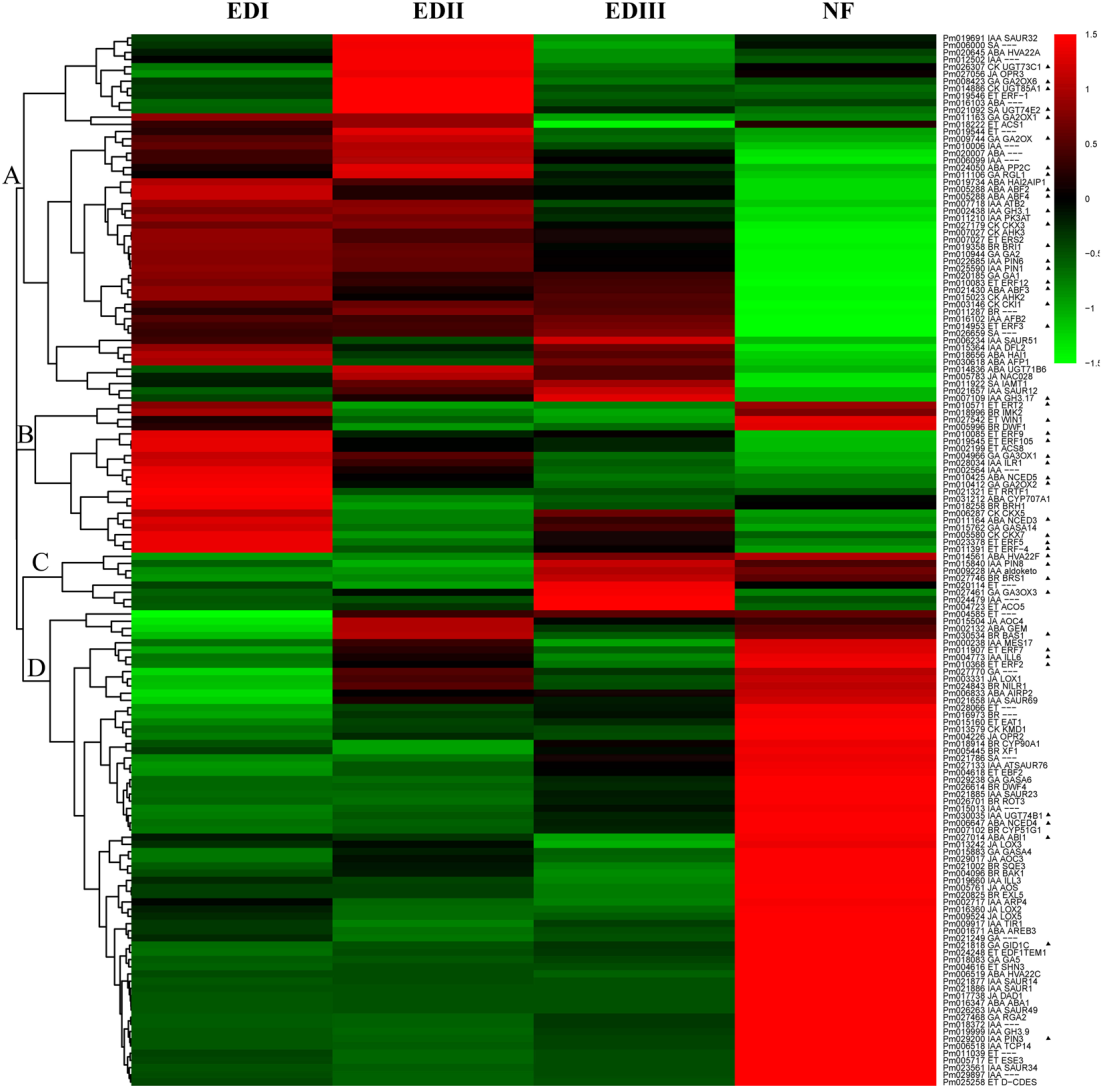
Hierarchical cluster analysis of hormone-related DEGs during the dormancy process in *P. mume*. Red indicates high relative gene expression and green indicates low relative gene expression. Letters assigned to major clusters are indicated on the dendrogram. Some key genes were highlighted with triangular symbol.

DEGs encoding hormone metabolism and signaling were further hierarchically clustered. As shown in Fig. 4, four main gene clusters were determined. Cluster A included 51 genes, some exhibiting their highest levels at EDI and some at EDII, and then gradually decreased as dormancy release progressed. ABA signaling genes, such as *ABF*2, *ABF3*, *ABF4* and *HAI1*, GA associated genes, such as *GA2OX1*, *GA2OX6*, *GA1* and *RGL1*, as well as with IAA-related genes, such as *ATB2*, *WAG1*, *GH3.1*, *GH3.17, PIN1*, *PIN6* and *DFL2*, all grouped within this cluster. *UDP-glycosyltransferases 71B6* (*UGT71B6)*, *UGT73C1* and *UGT74E2*, encoding UDP-glucosyl transferases that preferentially glucosylates ABA, CK and SA respectively, exhibited this expression pattern. Cluster B (21 genes) showed high expression level at EDI stage, and dramatically decreased at EDII, and then maintained relatively low level at EDIII and NF stages. Among them, *NCED3* and *NCED5* are key ABA biosynthesis genes, and *GA2OX2* is key GA catabolism gene, which are in agreement with high ABA and low GAs levels at EDI stage. Several ethylene response factors (*ERF*s), including *ERF2*, *ERF4*, *ERF5*, *ERF9*, *ERF105*, involved in ET-activated signaling pathway, also displayed this expression pattern. Cluster C contained 12 genes, which showed low expression levels at the EDI and EDII stages, then sharply increased at EDIII, and dramatically decreased at NF. *GA3OX3* and *ACO5*, keys genes involved in GA and ET biosynthetic process, and *BRS1*, involved in BR signaling pathway, belonged to this cluster. *PIN8*, encoding an auxin transporter, also displayed this expression pattern. Genes in Cluster D (66 genes) typically showed almost constant low expression levels from EDI to EDIII, and then sharply increased at the NF stage, when the dormancy was completely released. Among them, some key genes that respond to ABA (such as *NCED4*, *AREB3*), GA (*GA5*, *RGA2*, *GIDC*), IAA (*PIN3*, *ILL6*), BR (*BAS1*, *EXL5*), ET (*ERF7*, *EAT1*) and JA (*AOC4*, *LOX2*) were grouped within this cluster. *UGT74B1*, encoding a UDP-glucosyl transferase that preferentially glucosylates IAA, belonged to this cluster. More details can be seen in Supplementary Table S5.

### Cluster analysis of sugar-related DEGs during bud dormancy transition

**Figure 5:**
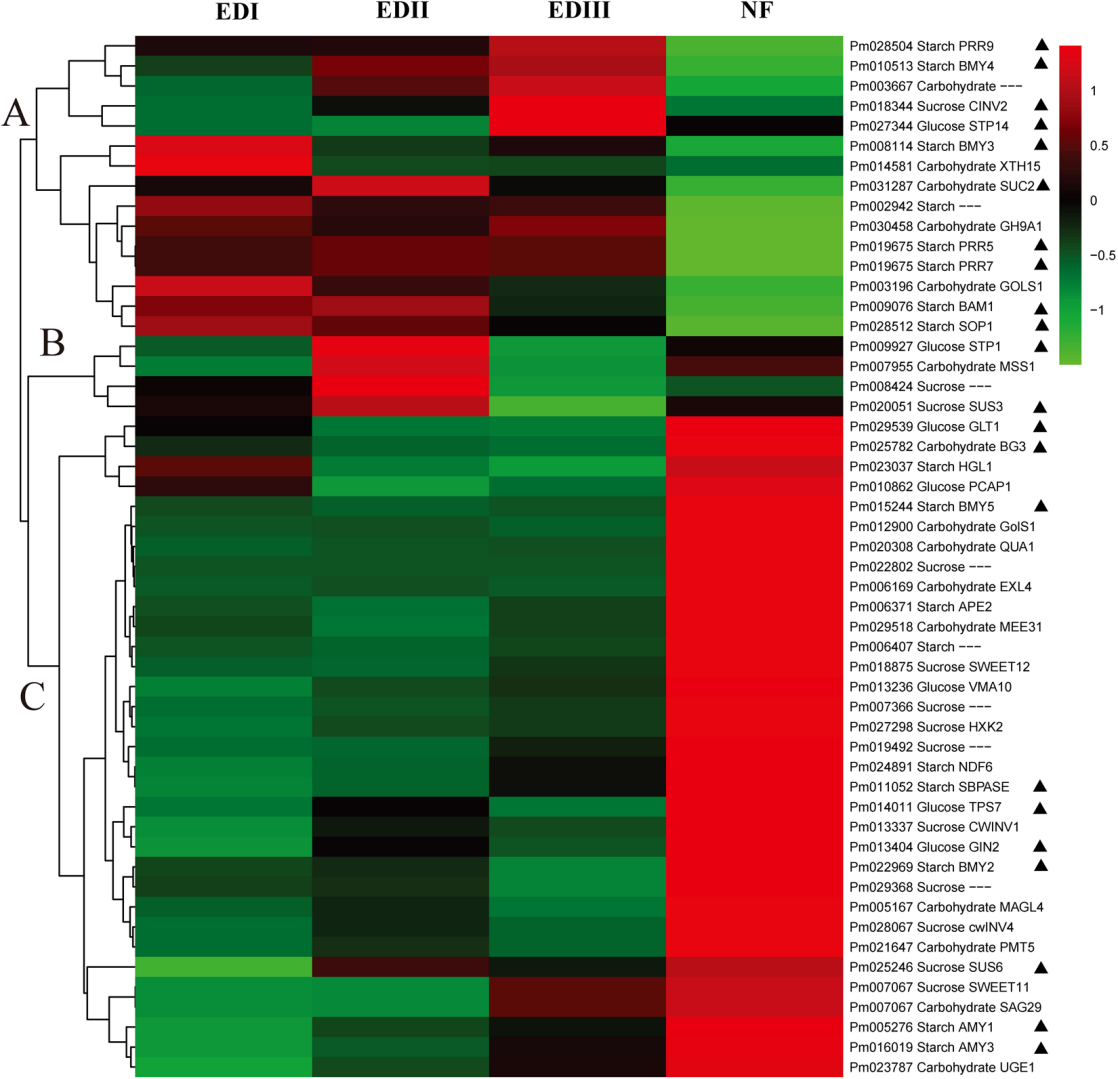
Hierarchical cluster analysis of sugar-related DEGs during the dormancy process in *P. mume*. Red indicates high relative gene expression and green indicates low relative gene expression. Letters assigned to major clusters are indicated on the dendrogram. Some key genes were highlighted with triangular symbol.

Sugar-related DEGs, including those involved in carbohydrate biosynthesis and metabolism, transportation and signaling, were grouped into three major clusters as shown in Fig. 5 and listed in Supplementary Table S6. In general, genes in Cluster A were expressed at high levels in at least one of the dormancy stages and at significantly lower levels at the NF stage. The high expression levels of *BMY3*, *BMY4*, *BAM1*, *pseudo-response regulator 5* (*PRR5*), *PRR7*, *PRR9* and *starch excess 1* in this cluster, the key genes in the starch metabolic process, are consistent with the decline in the starch contents. In addition, *sucrose*-*proton symporter 2* (*SUC2*), a major sucrose transporter, exhibited the same expression pattern. The genes in Cluster B (4 genes) exhibited a significant increase between the EDI and EDII stages and then decreased between the EDII and EDIII stages. Among them, major facilitator superfamily protein is involved in sugar transport, and *SUS3* is involved in sucrose biosynthesis. Group C, with the largest DEGs (33 genes), exhibited completely opposite expression profile with Cluster A, with a sharply increasing expression pattern during the dormancy release process. Among them, *beta-amylase 5* (*BYM5*), *BMY2*, *alpha*-*amylase*-*like* (*AMY1*), *AMY3* and *heteroglycan glucosidase 1* are involved in starch metabolic processes, and *sedoheptulose-bisphosphatase* and *NDH dependent flow 6* are involved in starch biosynthetic processes. Moreover, many of them are related to sucrose and glucose biosynthetic and metabolic processes, such as *sucrose synthase 6* (*SUS6*), *sucrose beta*-*fructofuranosidase*, *glucose insensitive* 2 (*GIN2*), and *trehalose*-*phosphatase*/synthase 7 (*TPS7*). In addition, *1,3-β-glucanase*, *UDP-glc 4-epimerase 1* and *maternal effect embryo arrest* 31, which involved in carbohydrate biosynthetic and metabolic process, also belong to this cluster.

### Cluster analysis of dormancy candidate genes during bud dormancy transition

**Figure 6:**
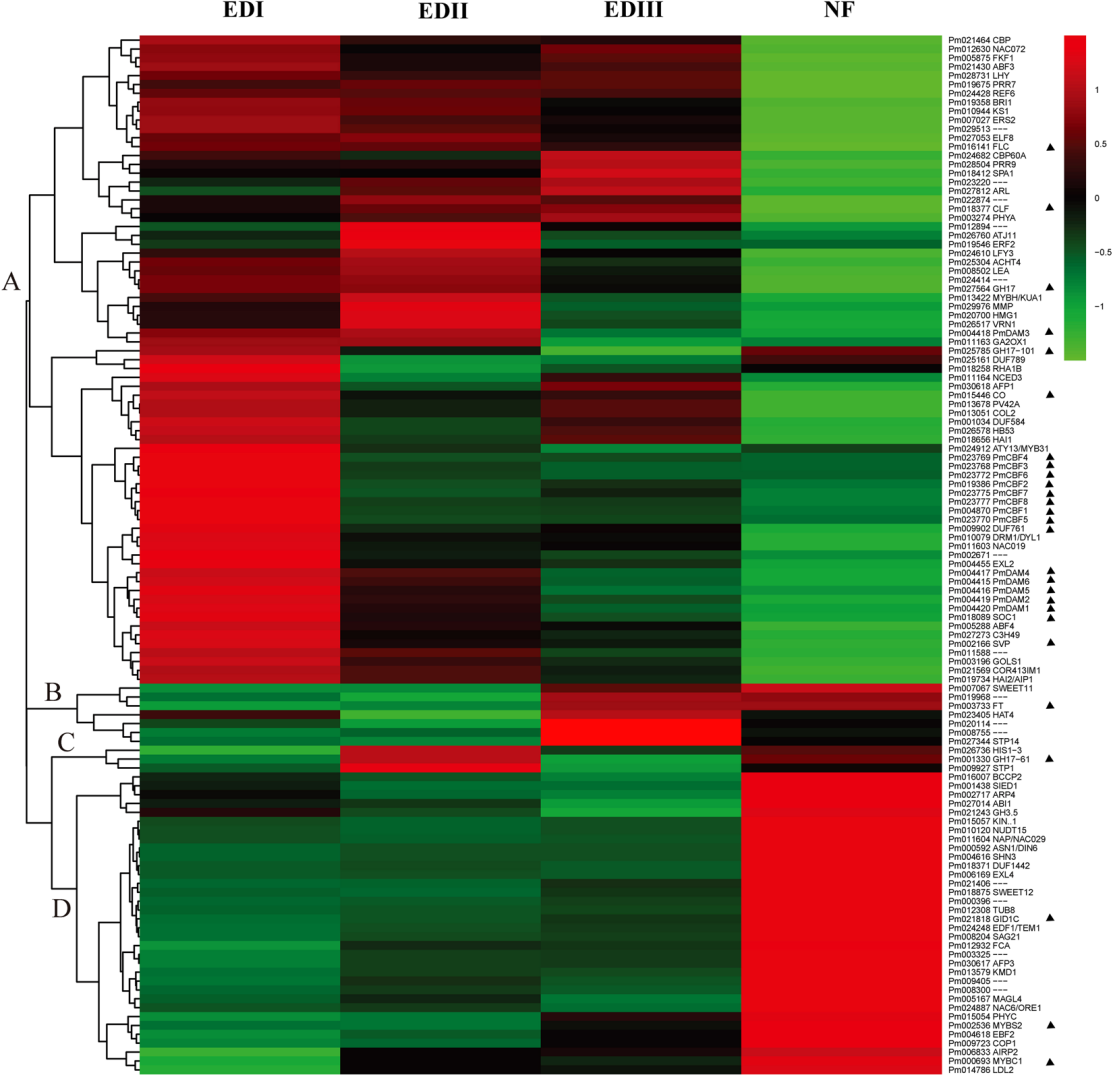
Hierarchical cluster analysis of 117 dormancy CGs during the dormancy process in *P. mume*. Red indicates high relative gene expression and green indicates low relative gene expression. Letters assigned to major clusters are indicated on the dendrogram. Some key genes were highlighted with triangular symbol.

In 1960, Chouard hypothesized that vernalization and dormancy development share similar signaling components and mechanisms^44^. Thereafter, evidence from various herbaceous and perennial species has directly or indirectly supported this hypothesis. Thus, 78 dormancy candidate genes (CGs) conserved in *Arabidopsis*^4^, and 79 CGs in the regulation of dormancy and flowering date in *Prunus* species, blackcurrant, poplar and *Arabidopsis*, were collected^45^. After strict homolog identification, 146 *P. mume* putative homologs were identified and 117 genes met the DEG criteria. Thus, only the 117 *P. mume* putative dormancy CGs were used for further analyses (Supplementary Table S7). Based on their expression profiles, the dormancy genes were analyzed using hierarchical clustering, and four major expression profiles were determined (Fig. 6). In general, genes in Cluster A displayed high expression levels in at least one of the dormancy stages and then sharply decreased at the re-active stage. Among these, 8 putative *PmCBF*s (named as *PmCBF1*–8 according physic locus) showed this expression pattern and all grouped together. MADS-box transcription factors, including *PmDAM1–6*, *short vegetative phase* (*SVP*), *suppressor of overexpression of CO 1* (*SOC1*), *flowering locuc C* (*FLC*), which are proven components in the dormancy process, exhibited this expression profile. *Glucan Hydrolase Family 17* (*GH17*) and *GH17*-101 regulating callose degradation, belonged to this cluster. Seven genes in Cluster B exhibited low expression levels at the EDI and EDII stages, and then sharply increased in the EDIII and NF stages. Of these genes, *FT* has been hypothesized as the essential component of the flowering network and many genes and signaling pathways converge on *FT*. *SWEET11* and *sugar transporter 14* (*STP14*), involved in carbohydrate transport, are grouped in this cluster. Cluster C (three genes) exhibited low expression levels at EDI and increased sharply at true endo-dormancy stage, and then decreased again at the EDIII and NF stages. *GH17*-61 and *STP1*, close paralogs of *GH17*-101 and *STP14* respectively, belonged to this cluster. Genes in Cluster D displayed consistent low levels in dormancy stages and much higher levels at the active stage. Among them, *KINβ1*, a subunit of the SnRK1 kinase, plays an important role in bud dormancy development by promoting sugar metabolism and cell division.

### Validation of RNA-Seq using qPCR

The total RNAs performed for RNA sequencing libraries were used as template for qPCR to validate the RNA-seq data. The qPCR of 12 hormone- and sugar-related genes at four dormancy stages showed the same trends as the RNA-Seq results, despite some differences in magnitude, suggesting that the RNA-Seq data in this study are reliable (Supplementary Fig. S6; Supplementary Table S8).

## Discussion

Bud dormancy is a complex programmed strategy that allows perennials to survive extreme low temperature in winter. As the chilling accumulation increases and favorable environmental conditions occur, the external signals are perceived by receptors, resulting in the generation of many secondary signaling molecules. Recently, expression profiles have been performed on several members of the *Prunus* genus. In this paper, to draw a complete dormancy-activity cycle, we reported a comprehensive transcriptome study at four critical developmental stages. We particularly focused on the MapMan BINs “major carbohydrate metabolism” and “hormone metabolism”, as well as their interaction.

The importance of hormone homeostasis in bud dormancy has been well reviewed^18^. ABA and GA are the core hormones that antagonistically regulate bud dormancy status. ABA and GA might interact and have both cause: ABA participates in the suppression of GA biogenesis^19^, and GA also negatively regulates ABA biogenesis during seed germination^20^. In the present investigation, the ABA content steadily decreased from EDI to EDIII, and even to NF (Fig. 2a). The GAs levels declined from EDI to EDII and then steadily increased (Fig. 2c–e). The ABA/GA ratio steadily decreased during the dormancy release process in our paper (Fig. 2f). The findings in this study regarding endogenous ABA/GA are consistent with those in grapes^24^, sweet cherry^25^ and tree peony^26^.

The decline in the ABA content might contribute to the decreased ABA biosynthesis genes and the increase of ABA catalysis genes. *Pm011164* and *Pm010425*, homologs of *ATNCED3* and *ATNCED5*, respectively, exhibited high expression levels during EDI and then sharply decreased during EDII (Fig. 4). *Pm031212* (homolog of *ATCYP707A1*) exhibited a high expression level at the EDI stage and then gradually decreased during the EDII stage, as confirmed by qPCR (Fig. 4 and Supplementary Fig. S6). Interestingly, *Pm014836* (homolog of *ATUGT71B6*) which can preferentially glucosylate ABA, was expressed at a high level during the EDII and EDIII stages, as confirmed by RNA-Seq and qPCR (Fig. 4 and Supplementary Fig. S6). Our results indicated that at the EDI stage, ABA might be hydrolyzed by *CYP707A1* into phaseic acid and/or dihydrophaseic acid. However, at the EDII and EDIII stages, active ABA was glucosylated by ABA glucociltransferase into ABA glucocil ester (ABA-GE), and stored in the flower buds and/or stems. Moreover, ABA-GE could be degraded by β-glucosidases (*BGs*) in only one step, if necessary^46^. To our surprise, *BG*s homologs in *P. mume* exhibited low expression levels (data not shown), suggesting that ABA-GE was not degraded by *BG*s during the bud dormancy release process in *P. mume*.

In the early stage of dormancy formation, the decrease in active GA levels was closely related to growth cessation. After enough chilling accumulation, these contents then increased^47^. In our study, the GA3 content at the EDI stage were slightly greater than at the EDII stage, and then, they sharply increased from the EDII to EDIII stage (Fig. 2c). This might contribute to the increased transcript abundance of GA3 biosynthesis genes and decreased GA3 catabolic genes. *Pm027461*, homologs of *ATGA3OX3*, respectively, a key GA biosynthesis gene, exhibited low expression levels at the EDI stage and then were sharply up-regulated at the EDII and EDIII stages (Fig. 4). *GA2OXs*, such as *Pm010412* and *Pm011163*, which deactivate bioactive GA, showed relatively high expression levels at the EDI stage and significantly low levels at the EDII stage (Fig. 4). These results were consistent with the decline in the GA contents from EDI to EDII and with the increase from EDII to EDIII.

The balance in ABA/GA signaling is also crucial for bud development. Shu *et al*.^20^ reported that *ABSCISIC ACID-INSENSITIVE 4* (*ABI4*) is a key factor that regulates seed dormancy by mediating the ABA/GA balance. *ABI4* positively regulates ABA catabolism by binding directly to the promoters of *CYP707A1* and *CYP707A2*, but no direct targeting of GA metabolism genes by *ABI4* has been detected. Nevertheless, in sorghum, *SbABI4* and *SbABI5* can directly bind to the promoter of *SbGA2ox3*, likely activating its expression and affecting seed dormancy^48^. *ABI3*, as the major downstream component of ABA signaling, is another main regulator of seed dormancy and germination^49^. Transgenic poplar overexpressing *ABI3*, do not form terminal buds, suggesting a role for ABA in bud dormancy establishment^50^. In our present study, the expression level of the *ABI3* homolog *Pm020417* was very low, suggesting that the ABA-signaling pathway was blocked during the dormancy release process (Fig. 4). Thus, the ABA/GA balance during biogenesis and/or their signaling levels may be essential for the bud dormancy–activity cycle in *P. mume*.

IAA is an essential hormone that is involved in almost all aspects of plant development and in processing environmental cues. For bud dormancy, the auxin-related mechanism remains largely unknown. Recently, Qiu *et al*.^51^ reported an increase in the IAA level and several enriched auxin-signaling pathways in the vascular cambium during the transition from dormancy to active growth in Chinese fir. El-Yazal *et al*.^52^ discovered that exogenous IAA could alter endogenous hormones and hasten bud break from dormancy in apple trees. Here, the IAA content was up-regulated from the dormancy to reactivity stages (Fig. 2b). Auxin response factors (*ARF*s), as transcriptional activators, play crucial roles in auxin metabolic pathways during bud dormancy in hybrid aspen^53^. The expression profiles of *ARF*s observed here indicated that Auxin signaling pathways might involve in the bud dormancy process of *P. mume*. Consistent with previous studies, there were up- and down-regulated *ARF*s during the same comparative periods, suggesting their potential roles are blurred.

In general, dormant buds are considered to be in an inactive state with limited metabolic activities, and the dormancy–activity transition process involved the extensive reconfiguration of carbohydrate metabolism^54^. In the present investigation, significant changes in sugar-related genes and content levels suggested that sugar might participate in and play important roles in the bud dormancy development of *P. mume*. Short day lengths and low temperatures during autumn triggered starch accumulations in stems and buds, and subsequently the stored starch was degraded to soluble sugars in response to freezing temperatures during winter^28^. An energy flow from source to metabolic sink was observed in our study. Starch and amylopectin levels were high in buds at the beginning of dormancy and then declined gradually until dormancy was completely released (Fig. 3a,b). Soluble sugar, sucrose and glucose levels showed the opposite dynamic pattern (Fig. 3c–e). These results are in agreement with those of leafy spurge crown buds during the dormancy transition process^29^. The high concentrations of sucrose and glucose improved the freezing tolerance by lowering the freezing point of free water and disrupting the formation of ice crystals^30^. This enables buds to survive winter. The hydrolysis of starch might also provide a carbon skeleton for the synthesis of needed amino acids, lipids and metabolites associated with bud growth as shown in Supplementary Fig. S5 and Supplementary Table S4.

In bud dormancy, the sucrose could promote bud release, not only as an energy supply, but also as a sugar signal that was independent from auxin signaling^32^. In the present research, the transcriptome data indicated that sugar metabolism and signaling are involved in bud dormancy development. The expression profiles of key genes involved in starch metabolism, and sucrose and glucose biosynthesis, such as *BMY5*, *BYM1*, *AMY1*, *AMY3*, *SUS3*, *SUS6* and *TPS7*, were consistent with a decrease in the starch and an increase in sucrose and glucose (Fig. 5). The genes involved in sugar transport, such as *GTL1*, *SUC2*, *PMT5*, *SAG29* and *MISS1*, as well as sugar signaling, such as *GIN2*, *PCAP1* and *BETAFRUCT4*, also increased with the dormancy release process. Similar results have been reported in other species, such as *Arabidopsis*^33^, poplar^55^ and grapevine^34^. Thus, these genes may be required for energy reconfiguration to acclimate to the external environment and survive cold winters.

Sugar alone, or through interactions with hormones, can induce or suppress many growth-related genes^56^. Additionally, crosstalk has been reported between sugar and hormones, including GA, ABA, IAA and SA. The α-amylase family is a relatively well-characterized example of interactions between hormone and sugar in dormancy process. α-Amylases act as key enzymes in starch degradation to generate soluble sugars, playing critical roles in the dormancy process^57^. They are tightly regulated by GA and ABA. In general, GA promotes the production of α-amylases by *GAMYB*, and ABA blocks the expression of α-amylases by an ABA-induced protein kinase (*PKABA1*) and two ABA-inducible WRKY proteins. PKABA1 and WRKY proteins suppress the GA-induced α-amylase’s expression by strongly inhibiting the expression of GAMYB and by competing with GAMYB, respectively^58–60^. In addition, SA might inhibit α-amylase production not by inhibiting α-amylase enzyme activities but by suppressing a GA-induced low pI α-amylase gene expression^57^. Moreover, SA and ABA promote *HvWRKY38* expression through an independent pathway and consequently, suppress the GA-inducible α-amylase further during seed dormancy process in rice^57^. Meanwhile, the application of exogenous glucose affects GA-mediated α-amylase expression and activity levels through complex pathways^61^. During the leafy spurge bud dormancy process, GA promotes the synthesis and activity levels of α-amylases, suggesting the potential for GA involvement in the breakdown of starch^62^.

Another example of sugar and hormone interactions involves the GA-inducible *GH17* family. During bud dormancy establishment, PD is blocked by callose, resulting in the inhibition of cell-to-cell communication^22^. Then, low temperatures induce the accumulation of 1,3-β-glucanases, which can hydrolyze callose. *GH17* is a relatively larger family, with 50 members in *Arabidopsis*, and groups into three clades (α, β and γ). Most members of α-clade localize to PD, while the γ-clade’s members localize to lipid bodies. Both of them can remove callose at PD and play crucial roles in the seed dormancy process^23^. In poplar, over 100 members were identified, and both α- and γ-clade members potentially target the PD’s callose^21^. In addition, 10 members are GA responsive and well investigated in relation to bud dormancy^21^. More importantly, three (*GH17*-44, *GH17*-61 and *GH17*) were mapped as CGs involved in bud dormancy and flowering time on different linkage groups constructed from two progenies of sweet cherry^45^. In our study, the high expression levels of *GH17*s (*Pm027564*, *Pm025785* and *Pm001330* homologs of *GH17*, *GH17-101* and *GH17-61*, respectively) and high contents of GAs suggest that they play crucial roles during the bud dormancy process of *P. mume* (Fig. 5).

Sugars can also affect hormone metabolism, and one mechanism is through UDP-glucose conjugation. UGTs are the most common enzymes that catalyze this process. UGTs can transfer UDP-glucose biosynthesized from sucrose through sucrose synthase to free hormones, resulting in conjugated inactive hormones^63^. At present, 107 *UGTs* have been identified in *Arabidopsis*^64^. Among them, eight UGTs were characterized to have ABA-associated UGT activity^65^, and five UGTs showed UGT activity for CK^66^. Free bioactive ABA in plant cells can be lowered in two ways, hydroxylation and conjugation. CYP707A1 hydroxylates ABA at the C-8´position and converts to phaseic acid and dihydrophaseic acid; on the other hand, UGT71B6 preferentially glucosylates ABA to form ABA-GE^67^. ABA-GE, as a stable storage form, contributes to ABA homeostasis because ABA-GE can be hydrolyzed through BGs in one step^46^. However, to our knowledge, the exact UGTs that can preferentially glucosylate GAs have not been identified. In our study, the dynamic patterns of several *UGTs*, such as *Pm014836* (*UGT71B6*, associated with ABA), *Pm030035* (*UGT74B1*, IAA), *Pm014886* (*UGT85A1*, CK) and *Pm026307* (*UGT73C1*, CK), suggested that hormone conjugation plays important roles during the dormancy process of *P. mume* (Fig. 4).

Recently, Niu *et al*.^16^ constructed a proposed *CBF*-*DAM-FT* model in pear. A hypothetical model on low temperature-induced *CBF*’s increasing freezing tolerance was proposed^68^. In brief, low temperature-induced *CBF* expression results in increased *GA20X* expression and the accumulation of *DELLA* proteins, which can reduce the GA content and block the GA signaling pathway, respectively. The overexpression of *CBF* genes can result in increased cold hardiness in several perennials, including poplar^69^, apple^70,71^ and grape^72^. Horvath *et al*.^73^ reported that *CBF* controlled cold-responsive *EeDAM1* by binding CBF sites in its promoter. In peach, CBF sites were found in the promoters of *PpDAM5* and *PpDAM6*, which were well-characterized within the dormancy process; however, no CBF sites were found in *PpDAM1*, *PpDAM2* or *PpDAM3*, which have not been associated with dormancy^8,74,75^. There is also some evidence that *DAM* regulates dormancy by inhibiting the flower-promoting *FT* genes^73^. The overexpression of *DAM1* in *Arabidopsis* resulted in decreased *FT* expression and delayed flowering time^73^. Chromatin immunoprecipitation assays indicated that *DAM* could bind to the CArG boxes in the promoter regions of *FT*^76^. Yeast one-hybrid and transient expression analyses demonstrated that *Pp*DAM1 could bind to the *PpFT2* promoter and inhibit its expression^16^.

In the *P. mume* genome, 13 putative *PmCBF* homologs and six tandemly arrayed *PmDAM*s were identified^35^. Among them, 8 genes were significantly changed during dormancy development. Surprisingly, all (*PmCBF1-8*) decreased during the dormancy release process, displaying the same pattern and grouped together as shown in Fig. 6. Moreover, all six *PmDAM*s were up-regulated more than twofold, and displayed similar dynamic patterns and grouped together, peaking at the EDI stage (Fig. 6). These results were consistent with other transcriptomic dormancy studies on peach^8,10^ and pear^11,77^. In the upstream regions of *PmDAM4*, 5 and 6, more CBF-binding sites were found than in peach. In *PmDAM1* and 6, one and two novel CBF sites were found, respectively, while *PmDAM2* and 3 had no CBF sites. Remarkably, the expression levels of these *PmDAM*s correlated with the number of CBF sites. *PmDAM4*, 5 and 6 (with several CBF sites) showed the highest levels, followed by *PmDAM1* (with one novel CBF sites) and then *PmDAM2* (with no *CBF* sites) (Fig. 6; Supplementary Table S7). Thus, dormancy-associated *DAM*s may be controlled by *CBF*s binding to the CBF sites in the upstream regions. In the present study, Given that *DAM5* and *DAM6* were well characterized as having dormancy-associated expressions, it is reasonable to hypothesize that these *DAM* genes might function in dormancy development in *P. mume*. In addition, other *MADS-box* genes, such as *SVP* (*Pm002166*), *SOC1* (*Pm018089*) and *FLC* (*Pm016141*), that are involved in bud dormancy displayed the same expression patterns as the *DAM*s and belonged to the same cluster (Fig. 6). These results suggested that *MADS-box* genes might be closely associated with the dormancy transition process in *P. mume*.

## Conclusions

**Figure 7:**
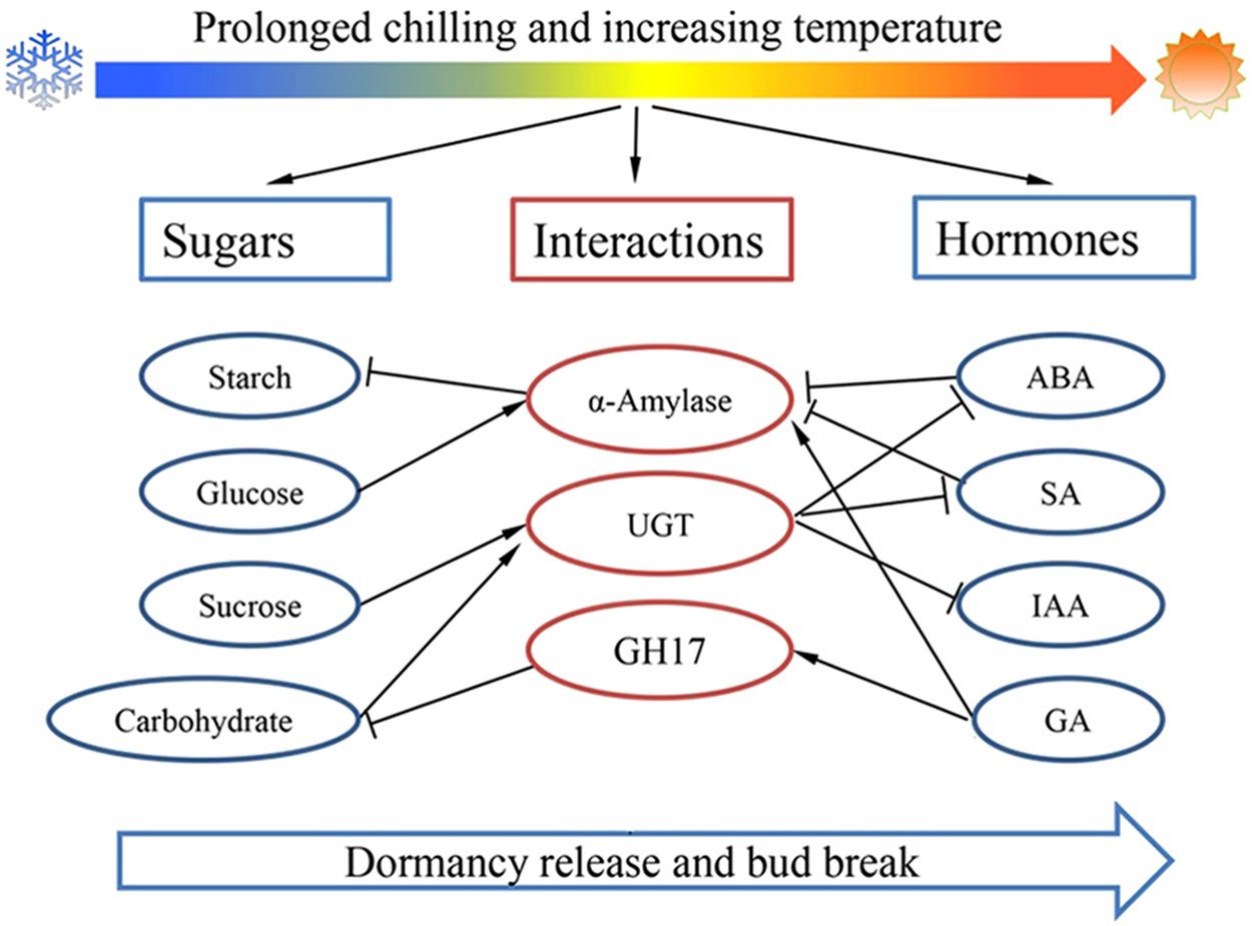
A hypothetical model for understanding the molecular mechanism of bud dormancy in *P. mume*. α-Amylase, *GH17* and *UGT* families might play crucial roles in the interactions between hormones and sugars. Positive and negative regulatory actions are indicated by arrows and lines with bars, respectively.

By combining the physiological and transcriptomic analyses, a hypothetical model was proposed for understanding the interactions between hormones and sugars (Fig. 7). During the four crucial dormancy stages, significant alterations in hormone contents and carbohydrate metabolism were observed, and α-amylase, *GH17* and *UGT* families may play crucial roles in the interactions between hormones and sugars. In autumn, low temperatures exposure activated the significant up-regulation of eight *PmCBF*s, and then, the *PmCBF*s blocked the GA-signaling pathway by accumulating DELLA proteins. In addition, the increased levels of *PmCBF*s activated the up-regulation of all six *PmDAM*s, resulting in growth cessation and dormancy establishment. Prolonged chilling and/or subsequently increasing temperature then reduced *PmCBF* expression levels, resulting in high levels of bioactive GAs and the reopening of GA pathways. The down-regulated *PmDAM*s removed the inhibition to *FT*. The high level of *FT* expression and the reopened growth-promoting GA-pathway promoted the dormancy release process and bud break under appropriate conditions.

## Methods

### Plant materials and dormancy status of flower buds

*P. mume* cultivar ‘Lve’ grown in the Jiufeng International Plum Blossom Garden, Beijing, China (40°07′N, 116° 11′ E) was used in this study. The temperature was recorded by HOBO U23-003 (Massachusetts, United States) every 30 minutes. A typical *P. mume* leaf axil has three separate buds, a single vegetative bud and two flower buds, and only the flower buds were sampled for further study. Flower buds were collected approximately every 7 d between 12 and 14 a.m. from 6 November 2015 to 8 March 2016 according to our multi-year field observation. Additionally, at every time point, 20 one-year-old shoots were randomly collected, and they were cultivated in water under the following phytotron conditions: 16/8 h 25/16 °C day/night at 70% relative humidity. The water was changed and the basal ends of the shoots were cut every 2 d. After 10 d, the dormancy status was evaluated as percentage bud break, which is the showing of a green tip. Flower buds on the cuttings were collected and stored at −80 °C until use.

### RNA extraction, Illumina sequencing, and quality control

For 12 bud samples, total RNA were extracted using the NanoDrop® 2000 (Thermo, CA, USA), and ~5 μg RNA was used for the RNA preparations. Sequencing libraries were generated using NEBNext^®^Ultra™ RNA Library Prep Kit for Illumina^®^(NEB, USA) and index codes were added to attribute sequences to each sample. The library preparations were sequenced on an Illumina Hiseq. 2500 platform and paired-end reads were generated following manufacturer’s recommendations. The sequencing reads were filtered using FastQC to remove primer/adapter and low-quality reads. The observed clean data with high quality were aligned to the *P. mume* genome, and then normalized into Reads Per Kilobase of transcript per million mapped reads (RPKM) values. DESeq R package (1.18.0) was used to analysis differential expression of two successive stages. The resulting p-value was corrected by Benjamini and Hochberg for controlling the false discovery rate (FDR). Differentially expressed genes (DEGs) were defined by FDR ≤0.05 and absolute fold change ≥1.5 (|log_2_Ratio| ≥0.58496) in successive stages.

### PCA, Venn and Heatmap diagrams

Principal component analysis (PCA) was performed using the online OmicShare tools (www.omicshare.com/tools). A Venn diagram of distribution of DEGs was constructed using the R package “VennDiagram”, and the Heatmap was constructed using the R package “pheatmap”.

### GO, KEGG and MapMan analyses

For all DEGs, functional annotation by gene ontology (Go) terms was analyzed using the Blast2GO program, with p-value ≤ 0.05 as significantly enriched. To analyze the main biological functions, we mapped the DEGs to terms in the KEGG (Kyoto encyclopedia of genes and genomes) pathways using R software. Mapman version 3.5.1 (http://mapman.gabipd.org/web/guest) was also used to identify specific enriched pathways. TAIR10 version (http://www.arabidopsis.org/) was used as reference with a P-value cut-off of ≤0.05 as ontology.

### RNA-Seq validation using real-time quantitative-PCR (qPCR)

The cDNA was synthesized from ~2 μg of total RNA for each sample. The gene-specific primers were designed at the website (http://sg.idtdna.com/primerquest/Home/Index), and the specificity was tested using Primer-BLAST (https://www.ncbi.nlm.nih.gov/tools/primer-blast/index.cgi?LINK_LOC=BlastHome). Protein phosphatase 2A (*PP2A*, *Pm006362*) was used as the internal reference control. The analysis was performed with three biological replicates. Fold change was calculated using standard 2^−ΔΔCT^ method. Twelve hormone-related and sugar-related genes qPCR primers are listed in Supplementary Table S8.

### Measurements of sugar and hormone contents

Dry and/or fresh buds at four developmental stages were used for sugar and hormones extraction and determination. Dry buds (~0.3 mg) were used for starch and amylose extractions. The starch contents were measured as described by Rosa *et al*.^78^, and the amylose contents were determined with an amylose measurement kit (A152-1) from Nanjing Jiancheng Bioengineering Institute (Nanjing, Jiangsu, China). For soluble sugar, sucrose and glucose extractions, ~0.5 g fresh buds were used, and they were quantified using a soluble sugar kit (A145), sucrose measurement kit (A099-1) and glucose assay kit (F006), according to the manufacturer’s instructions, respectively. Meanwhile, ~1 g fresh buds were used for ABA, IAA, GA1, GA3 and GA4 extractions with three biological replicates. The hormonal quantification was carried out using HPLC–ESI–MS/MS with a standard measure as described by Dobrev and Vankova^79^ and Djilianov *et al*.^80^.

### Bud candidate gene selection and the identification of *P. mume* orthologs

Based on available dormancy-related data in herbaceous and woody species, 78 dormancy candidate genes conserved in *Arabidopsis* published by Tarancón *et al*.^4^, and 79 CGs conserved in *Prunus* species, blackcurrant, poplar and *Arabidopsis* published by Castède *et al*.^42^ were selected (Supplementary Table S8). For each CG, the peach ortholog was identified from *Arabidopsis* sequence in Phytozome v12 (https://phytozome.jgi.doe.gov/pz/portal.html) and other published studies. The putative *P. mume* orthologs were identified by BLASP (E-value < 1e-10, identity >30% and coverage >70%) using *Arabidopsis* and peach sequences as query, and genes with the highest E-values were selected. As the well-characterized strong CGs, all 13 putative *PmCBF*s and 6 *PmDAM*s identified in *P. mume* genome^19^ were included for further analysis. Furthermore, a Pfam domain analysis (http://pfam.xfam.org/) was performed for each CG to ensure the accuracy of the orthologs in three species. In addition, phylogenetic trees were built using MEGA7.1 (Maximum-likelihood method, 1,000 bootstrap replicates) to identify the most closely related orthologs.

### Statistical analysis

For hormone, sugar and qPCR results, statistical analysis was performed using SPSS version 19 (SPSS, Chicago, IL, USA) with ANOVA and Duncan’s test. Data are means with three biological replicates each, with error bar representing standard error. Non-overlapping letters (a–d) indicate significant differences between different bud stages, based on analysis of variance and multiple range test procedures with a confidence level of 95%.

### Data availability

All data generated or analyzed during this study are included in this published article (and its Supplementary Information files) and also are available from the corresponding author on reasonable request.

## Electronic supplementary material

Supplementary information

Dataset 1

Dataset 2

Dataset 3

Dataset 4

Dataset 5

Dataset 6

Dataset 7

Dataset 8
