# Supplementary material for: Transcriptome Profiles Reveal the Crucial Roles of Hormone and Sugar in the Bud Dormancy of Prunus mume
Source: Sci Rep. 2018 Mar 23;8:5090. doi: 10.1038/s41598-018-23108-9 (PMC5865110; doi:10.1038/s41598-018-23108-9)
Supplement: Supplementary file 1 — Supplementary information [file 41598_2018_23108_MOESM1_ESM.pdf]

Supplementary information

Original Research Article

Transcriptome Profiles Reveal the Crucial Roles of Hormone and Sugar in the Bud Dormancy of *Prunus mume*

Zhiyong Zhang, XiaoKang Zhuo, Kai Zhao, Tangchun Zheng, Yu Han, Cunquan Yuan and Qixiang Zhang*

Beijing Key Laboratory of Ornamental Plants Germplasm Innovation & Molecular Breeding , National Engineering Research Center for Floriculture , Beijing Laboratory of Urban and Rural Ecological Environment , Key Laboratory of Genetics and Breeding in Forest Trees and Ornamental Plants of Ministry of Education , School of Landscape Architecture , Beijing Forestry University, Beijing, 100083, China

***Correspondence:**
Qixiang Zhang
zqxbjfu@126.com

Supplementary Table legends

Supplementary Table S1: The total information of the 5831 DEGs at four dormancy stages.

Supplementary Table S2: Gene ontology enrichment analysis of all 5831 DEGs. Gene sets that were significant at p-value < 0.05 are presented in table.

Supplementary Table S3: Signaling pathway enrichment analysis of all 5831 DEGs.

Supplementary Table S4: MapMan Bins of "Metabolism_overview" and "Regulation_overview" between two successive stages.

Supplementary Table S5: Detail information of hormone-regulated DEGs.

Supplementary Table S6: Detail information of sugar-regulated DEGs.

Supplementary Table S7: Detail information of dormancy candidate genes.

Supplementary Table S8: Primers information used in qPCR analysis

Supplementary Figure legends

Supplementary Figure S1: PCA analysis of the 12 transcriptome data of *P. mume*. Samplings were under four different conditions: EDI, EDII, EDIII and NF.

Supplementary Figure S2: Venn diagram of 5831 DEGs between two stages. Analysis was under four different conditions: EDI, EDII, EDIII and NF.

Supplementary Figure S3: Gene ontology categories of DEGs of *P.mume*. Analysis was under four different conditions: EDI, EDII, EDIII and NF. (**a**) EDI vs. EDII; (**b**) EDII vs. EDIII; (**c**) EDIII vs. NF. Up-regulated genes are illustrated in red and down-regulated genes are in green.

Supplementary Figure S4: KEGG pathway categories of DEGs of *P.mume*. Analysis was under four different conditions: EDI, EDII, EDIII and NF. (**a**) EDI vs. EDII; (**b**) EDII vs. EDIII; (**c**) EDIII vs. NF.

Supplementary Figure S5: A global view of metabolism and regulation changes for three successive stage comparisons with MapMan analysis. (**a**) "Metabolism_overview" of EDI vs. EDII; (**b**) "Metabolism_overview" of EDII vs. EDIII; (**c**) "Metabolism_overview" of EDIII vs. NF; (**d**) "Regulation_overview" of EDI vs. EDII; (**e**) "Regulation_overview" of EDII vs. EDIII; (**f**) "Regulation_overview" of EDIII vs. NF. Up-regulated genes are illustrated in red and down-regulated genes are in green.

Supplementary Figure S6: Expression detection of selected transcripts by RNA-Seq and qRT-PCR. Analysis was under four different conditions: EDI, EDII, EDIII and NF.

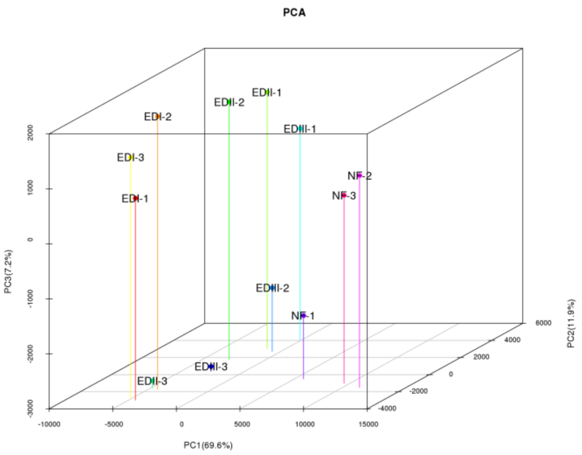

Figure S1 PCA analysis of the 12 transcriptome data of *P. mume*. Samplings were under four different conditions: EDI, EDII, EDIII and NF.

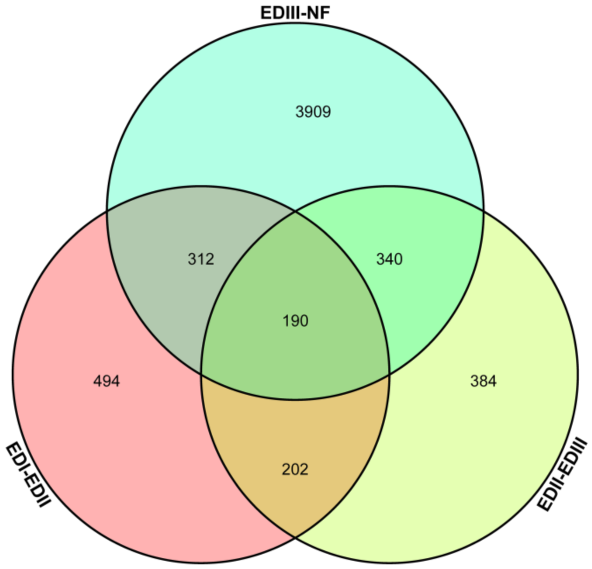

Figure S2 Venn diagram of 5831 DEGs between two stages. Analysis was under four different conditions: EDI, EDII, EDIII and NF.

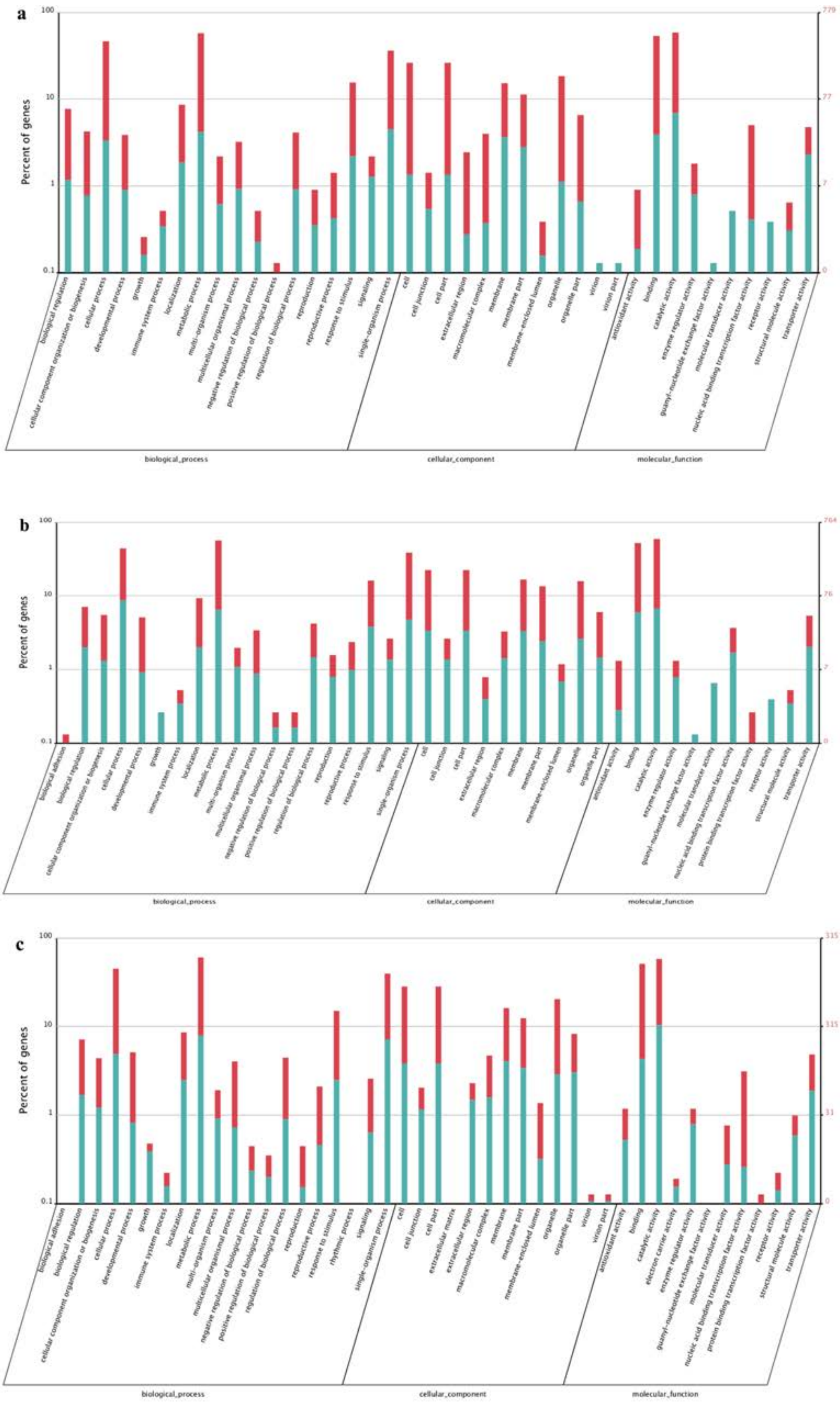

Supplementary Figure S3: Gene ontology categories of DEGs of *P.mume*. Analysis was under four different conditions: EDI, EDII, EDIII and NF. (**a**) EDI vs. EDII; (**b**) EDII vs. EDIII; (**c**) EDIII vs. NF. Up-regulated genes are illustrated in red and down-regulated genes are in green.

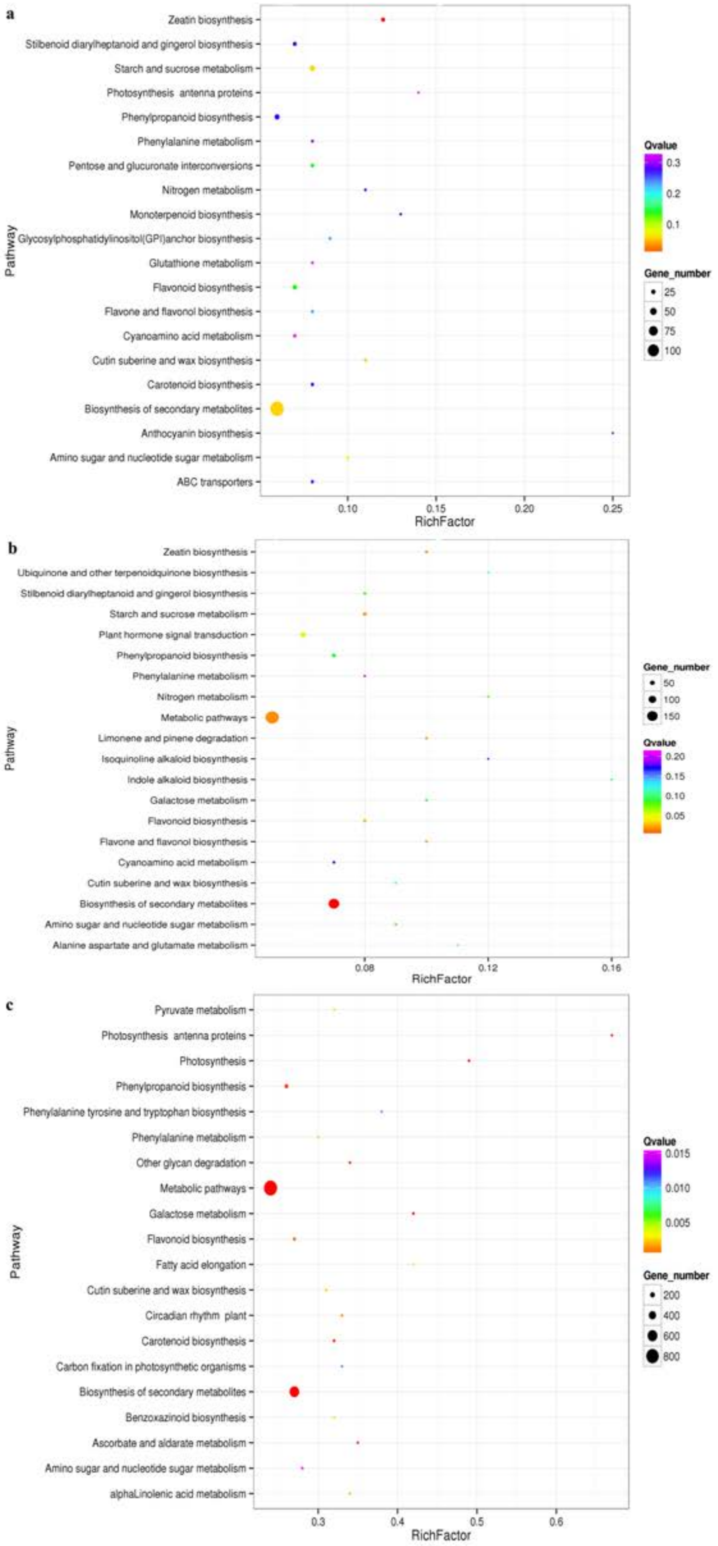

Supplementary Figure S4: KEGG pathway categories of DEGs of *P.mume*. Analysis was under four different conditions: EDI, EDII, EDIII and NF. (**a**) EDI vs. EDII; (**b**) EDII vs. EDIII; (**c**) EDIII vs. NF.

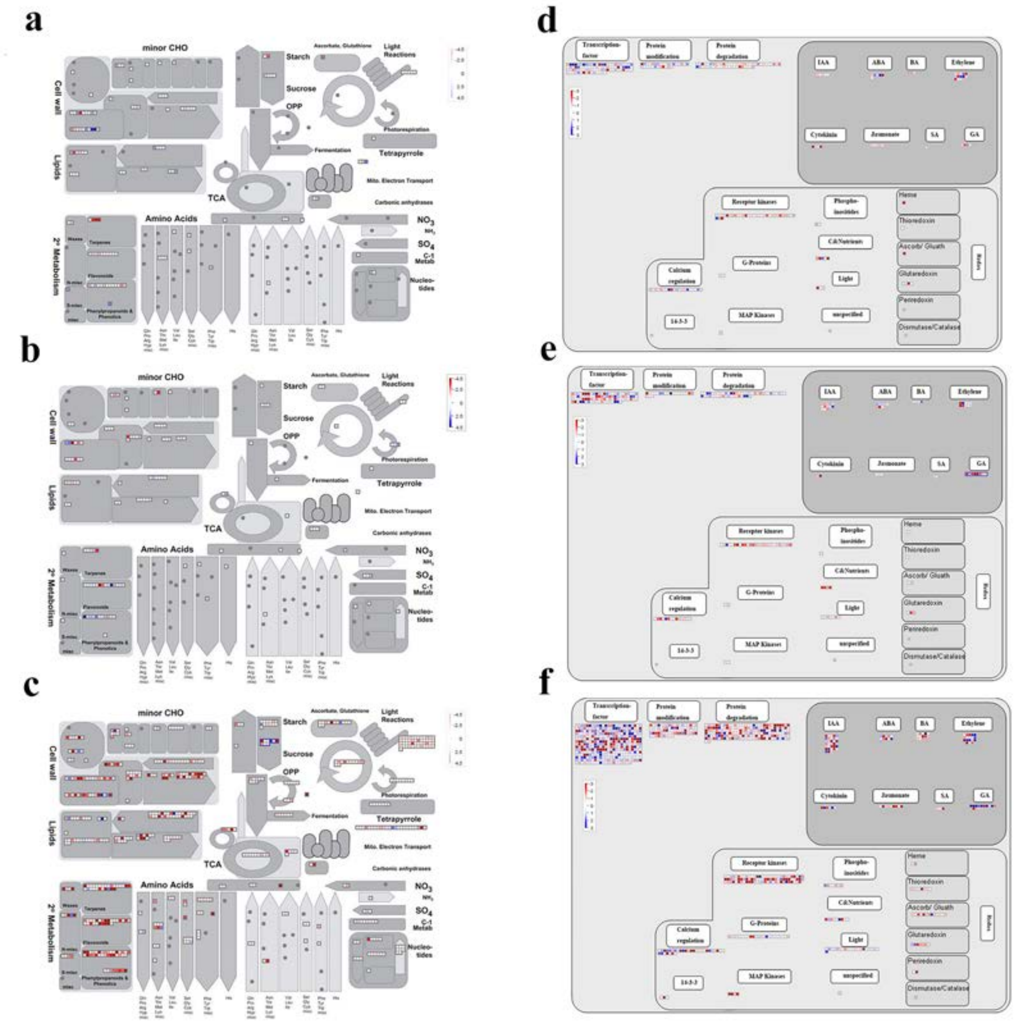

Supplementary Figure S5: A global view of metabolism and regulation changes for three successive stage comparisons with MapMan analysis. (**a**) "Metabolism_overview" of EDI vs. EDII; (**b**) "Metabolism_overview" of EDII vs. EDIII; (**c**) "Metabolism_overview" of EDIII vs. NF; (**d**) "Regulation_overview" of EDI vs. EDII; (**e**) "Regulation_overview" of EDII vs. EDIII; (**f**) "Regulation_overview" of EDIII vs. NF. Up-regulated genes are illustrated in red and down-regulated genes are in green.

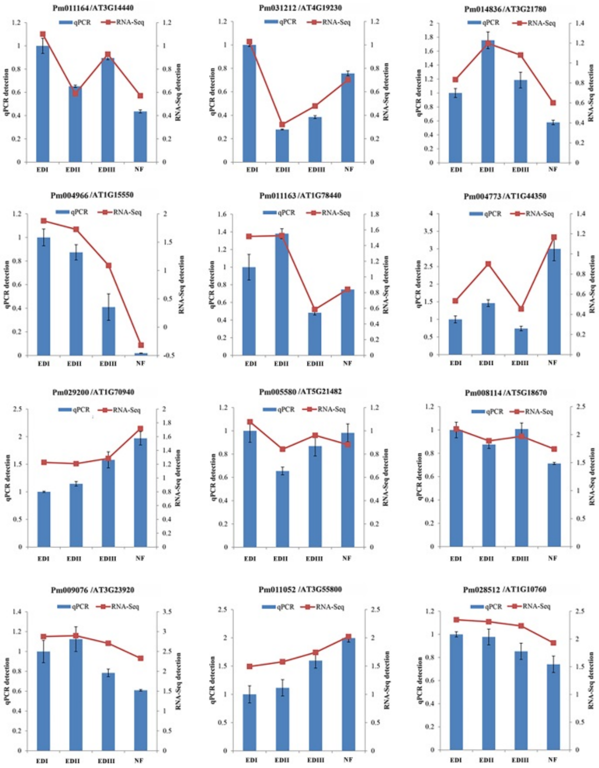

Supplementary Figure S6: Expression detection of selected transcripts by RNA-Seq and qRT-PCR. Analysis was under four different conditions: EDI, EDII, EDIII and NF.
